# Supplementary material for: Distinct Fecal Proteolytic Activity in Zoo Animals with Different Feeding Strategies
Source: Animals (Basel). 2025 Dec 11;15(24):3559. doi: 10.3390/ani15243559 (PMC12730040; doi:10.3390/ani15243559)
Supplement: Supplementary file 1 [file animals-15-03559-s001.zip › animals-3884345-supplementary.pdf]

Supplementary data

Animal	Sample	A-A	C-C	D-D	E-E	F-F	G-G	H-H	I-I	K-K	L-L	M-M	N-N	P-P	Q-Q	F-R	R-R	S-S	T-T	V-V	W-W	Y-Y
Tiger	1					153			6	4096	52	12				8088	4529				15	813
	2			5		138				3825	68	7		6		9821	4306				14	793
	3			16		181		14	13	2006	373	53				9242	2992	12	23	10	31	949
	4			8		97			7	2354	285	21				7312	2010		7		27	632
Lion	1			10		17				456	21					253	522				10	160
	2					126				826	93					602	873				39	931
	3					275			7	736	208	25				8094	494				112	1300
	4	10				130				450	241	19				5136	57		15	9	79	1097
Slow loris	1									317	21					10487	194					42
	2									474						2408	772					
	3	19		12						502	10			5		527	835	18	7			6
Meerkat	1					293			8	5244	256	49		8		10251	3939	5			12	814
	2					238			8	5077	311	29		7		9219	4127	8			6	992
	3					140			6	2960	108	7				8879	2091					564
	4					36				3532	81					2838	2597					352
Otter	1			10		249			11	4577	405	95				4303	3560			9	5	1032
	2			12		131			7	3914	319	81				5456	2020			5		721
	3			28		148			10	2772	292	106				2788	1721					620
	4			31		276		16	15	1998	211	395				6414	2963		30	43	29	519
Fishing cat	1					70				366	173	11				6799	86				124	630
	2	16	7			161		32	11	104	167	78	12			9996	79		22	9	434	337
	3					138		21	11	117	165	55	10			5508	75	5	7	7	408	894
	4	15				227		21	13	107	222	50	10			7489	66		17	9	313	591
Badger	1			15		172			5	284	196	20				10061	65			6	129	989
	2					457			12	739	216	46				2417	495				115	910
	3					702		6	16	403	145	103				10429	172				119	714
	4			11		30		6		166	70	34				8762	259		6		313	836
Raccoon	1	5				134			8	870	110	25				10197	85				210	752
	2	8				145		5	11	915	161	22				9411	87				155	623
	3			11		63			7	1633	110	26				9583	139				164	646
	4					38				1366	117	8				3903	326				73	725
Pig	1									80						47	129					13
	2									96						43	143					6
	3									109						57	154					9
	4									59						35	97					
Giraffe	1									79	6					103	54					
	2									89						116	56					
	3									53						32	30					
	4									86	26	19				53	37					6
Camel	1									646				9		889	417					
	2									635						582	426					5
	3									502						608	444					
	4									455						252	690					
Zebra	1									13						8	21					
	2									12							15					
	3									13						7	17					
	4									20							28					
	5									27						18	40					
	6									27						10	41					
	7									17	9					29	19					
Elephant	1									34						19	34					
	2									17						15	25					
	3									27						20	27					
	4									22						7	26					
Lemur	1									303	10					328	411					6
	2									302						199	517					
	3									216						227	421					
	4	6								219	5			5		330	402	8				5
Fruit bat	1					11				868	15			7		7041	99				45	357
	2									252						151	416				7	64
	3	5	8			169		32	15	886	106	46				9660	140		5		194	451

Supplemental Figure S1. Heat map of the 21 FRET peptide substrates screened with feces from zoo animal species with different diet requirements. Color scheme: black = lack of activity (F/min < 5); light green = low activity (F/min = 5 – 50); medium green = moderate activity (F/min = 51 – 500); dark green = high activity (F/min > 500).

Supplemental Table S1. Characteristics of the animals from which fecal samples were collected in this study.

	Animal species	No. of animals in the enclosure	No. of fecal samples collected	Provided diet	Sub-group †	Origin
Carnivore	Tiger (*Panthera tigris altaica*)	2	4	Rabbit, horse meat and beef, goat, goose, chicken	n.a.	Dierenpark Amersfoort
	Lion (*Panthera leo*)	3	4	Rabbit, horse meat and beef, goat, goose, chicken	n.a	Dierenpark Amersfoort
	Slow loris (*Nycticebus pygmaeus*)	4	3	Mealworm, lori nectar, vitamin powder, endive, broccoli, chicory, cricket, morio worm, wax moth larvae	Insectivore	Dierenpark Amersfoort
	Meerkat (*Suricata suricatta*)	7	4	Omnivore pellet, chicory, cricket, egg, mealworm, mice, wax moth larvae, day-old chicken, morio worm, cockroach	Insectivore	Dierenpark Amersfoort
	Asian small clawed otter (*Aonyx cinerea*)	4	4	Roach, cat pellet, crab, shrimp, snail, fish-eater-supplement	Piscivore	Dierenpark Amersfoort
	Fishing cat (*Prionailurus viverrinus*)	2	4	Rabbit, guinea pig, freshwater fish, rat, mice	Piscivore	Diergaarde Blijdorp
Omnivore	Raccoon (*Procyon lotor*)	6	4	Small fish, chicken, egg, herring, noodle bowl of mussels with shell, omnivore pellets, mix of vegetables, fruits, insects, nuts, and seeds	n.a.	Diergaarde Blijdorp
	Badger (*Meles meles*)	2	4	Mice, earthworm, walnut, mealworm,	n.a.	Dierenpark Amersfoort

				sunflower seeds, day-old chicken, morio worm, quail		
	Bentheim black pied pig (*Sus scrofa domesticus*)	2	4	Celeriac, parsnip, endive, pig pellets	n.a.	Dierenpark Amersfoort
Herbivore	Giraffe (*Giraffa camelopardalis*)	4	4	Boskos, pellet, alfalfa hay, fresh browse	Ruminant/ Fore-gut fermenter	Dierenpark Amersfoort
	Camel (*Camelus bactrianus*)	3	4	Alpaca pellets, grass hay	Ruminant/ Fore-gut fermenter	Dierenpark Amersfoort
	Zebra (*Equus grevyi*)	3	7	Herbivore pellets, grass hay	Non-ruminant/ Hind-gut fermenter	Dierenpark Amersfoort
	Elephant (*Elephas maximus*)	5	4	Elephant pellets, beetroot, chicory, training biscuit, grass hay, straw, fresh browse	Non-ruminant/ Hind-gut fermenter	Dierenpark Amersfoort
	Ring-tailed lemur (*Lemur catta*)	11	4	Primate leaf-eater pellet, tomato, broccoli, celery, turnip, red pepper, endive	Frugivore	Dierenpark Amersfoort
	Fruit bat (*Rousettus aegyptiacus*)	170	3	Tomato, apple, pepper, bat nectar	Frugivore	Dierenpark Amersfoort

† n.a.: not applicable.
